# The characteristics of combustion reactions involving thermite under different shell materials

**DOI:** 10.1039/d0ra05415a

**Published:** 2020-09-21

**Authors:** Jialin Chen, Tao Guo, Jiaxing Song, Miao Yao, Wen Ding, Xiaofeng Liu, Rui Zhu

**Affiliations:** a College of Field Engineering, PLA Army Engineering University Nanjing 210007 China; b School of Chemical Engineering, Nanjing University of Science and Technology Nanjing 210094 China; c School of Mechanical and Aerospace Engineering, Nanyang Technological University 639798 Singapore

## Abstract

To study the influence of tubular shell materials on the combustion of thermite, numerical simulations and experimental comparisons of the combustion efficiencies of thermite with PVC and stainless-steel shell materials were carried out. The thermal conductivity coefficient and heat radiation correlation coefficient of a shell material directly affect heat transfer during a heat-transfer process, that is, the lower the thermal conductivity and the higher the heat radiation reflectance coefficient, the lower the heat flux through the material and the less heat is lost. The experimental results show that compared with the stainless-steel tube material, the temperature distribution of thermite is more concentrated and the effect of melting through a steel target plate is more apparent when PVC is used as the shell material. The simulation results show that thermite in the PVC shell can produce a higher temperature, reaching 2200 °C at the loading port and 1700 °C on the steel target plate, which is maintained for 0.9 s. However, the corresponding maximum temperatures for the stainless-steel shell are only 2000 °C and 1500 °C, not yet reaching the melting point of the steel plate. The simulation results are consistent with the experimental phenomena. This work is of great significance for improving the design of thermite shells, enhancing performance, and guiding future combustion process research.

## Abstract 2

To study the influence of tubular shell materials on the combustion of thermite, numerical simulations and experimental comparisons of the combustion efficiencies of thermite with PVC and stainless-steel shell materials were carried out.

## Graphical abstract

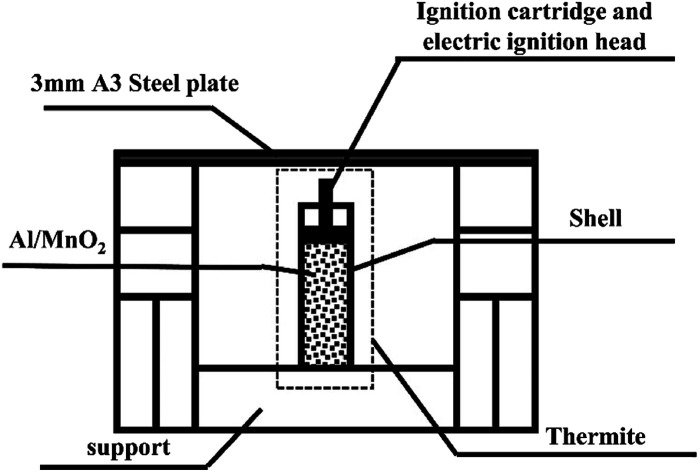

## 1. Introduction

Thermite, an energetic material with excellent combustion performance, releases lots of heat during the burning process^1–3^ and can produce large amounts of energy. It is widely used in many practical projects, such as combustion devices, rocket thrusters, and fuel–air explosives.^4–8^

In recent years, a lot of research into thermite has been conducted by scholars. Song and co-authors^9^ studied the effects of aluminum particle size on the thermal performance and kinetics of an Al/MnO_2_ thermite system. Ouyang Dehua and Pan Gongpei *et al.*^10^ measured the thermodynamic properties of flame-type thermite and provided the thermodynamic parameters. Fathollahi and co-authors^11^ studied the thermal behavior and kinetics of pyrotechnic compositions of Al + KClO_4_, Mg + KClO_4_, Al + Mg + KClO_4_, MgAl + KClO_4_, and Al + MgAl + KClO_4_ mixtures. The results showed that the composition containing MgAl had the highest activation energy and frequency factor. However, the critical ignition temperature of its oxidation reaction was lowest. Shepherd *et al.*^12^ studied the reactivity characteristics of a Si–BaSO_4_ composition and compared it with Si–CaSO_4_. It was found that compared with BaSO_4_ compositions, CaSO_4_-based formulations tend to have higher energy outputs and higher transient pressures. Wang and co-authors successfully prepared Al/CuO and Si/CuO nanothermite with a core–shell structure *via* a self-assembly method and electrophoretic deposition method, which showed excellent thermal performance.^13–15^ Mao and co-authors^16^ used F_2311_ as a binder and successfully prepared Al/CuO nanothermite through novel DIW 3D printing technology using nanothermite ink with good rheological shear thinning properties, whose burning rate increased significantly. So far, in terms of formula selection and structural optimization, many studies have been carried out on how to improve the combustion performance of thermite but, for specific applications, more research is needed.

A. G. Merzhanov and V. M. Shkiro^17^ combined thermal explosion theory with the ignition mechanism of an Al/Fe_3_O_4_ system to study the ignition process of thermite. Gibson and Lowell *et al.*^18^ also examined the relationship between the ignition temperature and the thickness of the Al_2_O_3_ film on the surface of the Al component in an Al/Fe_3_O_4_ system and concluded from experiments that the ignition temperature had a positive correlation with the thickness of the oxide film; this laid a certain foundation for studying the effects of the degree of Al oxidation on thermite. These studies theoretically analyzed how to improve the combustion effect based on the ignition mechanism. However, through a large number of experiments, we found that the use of different shell materials would produce different effects from identical thermite samples. What is more, there was a certain regularity to the effects observed.

Besides, with the development of applications, such as fireworks and propellants, that use thermite as a fuel, corresponding products will be produced and applied in the future. Then, it will inevitably be considered which materials should be used as restraints. Therefore, according to the actual future applications of thermite, it is of great significance to explore the influence of different shell materials on the combustion of thermite. In this paper, the effects of different tubular shell materials on the combustion properties of flame-type thermite were explored. Experiments involving thermite melting through a steel target plate with a PVC charge shell and stainless-steel material charge shell were designed. Based on the design of the experiments, a fluid–solid heat transfer model was theoretically established, and the burning heat transfer process of thermite was deduced and analyzed. The model was simulated using software. Finally, the effects of the shell materials on the combustion performance were discussed.

## 2. Experimental

### 2.1 Experimental design

A steel target plate with a thickness of 3 mm was used for penetration testing.

**Fig. 1:**
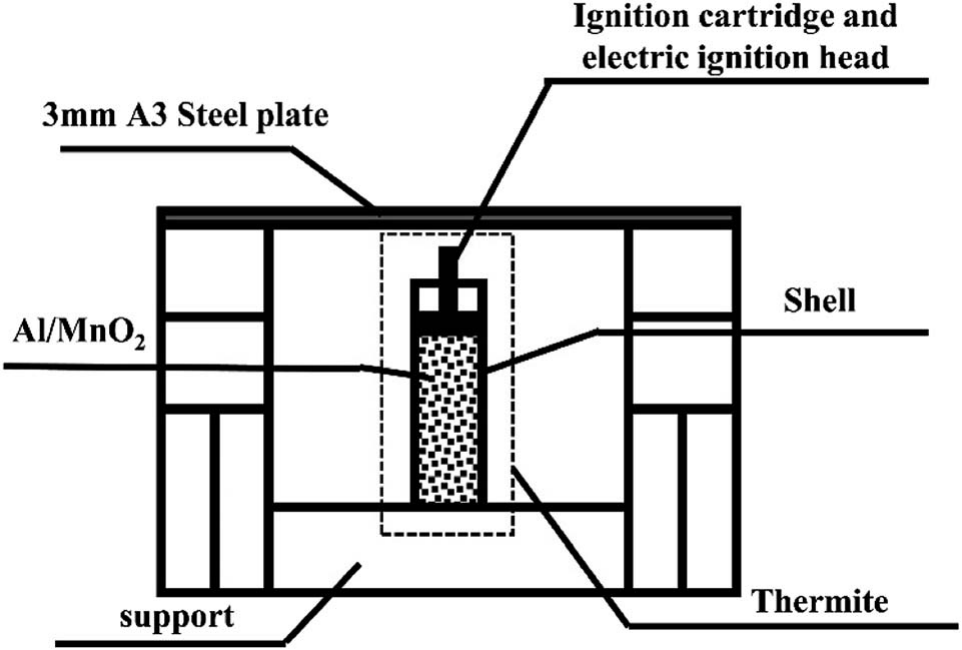
A schematic diagram of the set-up where thermite melted through a steel target.

A schematic diagram of the experimental device is shown in Fig. 1.

Stainless steel has good thermal conductivity, high density, and low heat radiation reflectance, significantly different from PVC. Besides, considering the ease of material processing, we chose stainless steel and PVC, which are representative of metal and plastic materials. Therefore, the thermite shells were made of PVC and stainless steel, respectively. The thermite consists of an Al/MnO_2_ thermite charge, whose formulation ratio is reported in a previous article.^19^ The middle of the charge port directly faces the 3 mm A3 steel target plate, which is about 3 cm above the thermite, so that the thermite flame can completely act on it. As it is ignited utilizing electric ignition, an ignition pillar and an electric ignition head are inserted into the thermite.^20^

**Fig. 2:**
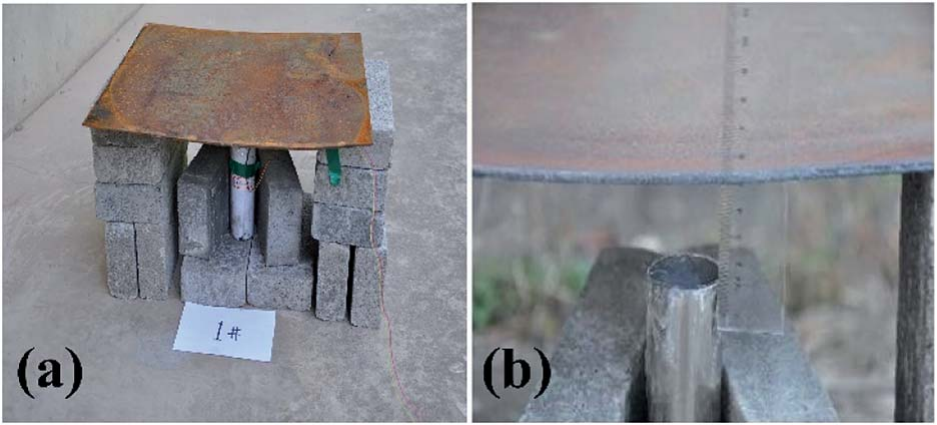
Photographs of the experimental device where thermite melts through a steel target plate: (a) the overall experimental device; and (b) a partially enlarged view.

Fig. 2 shows photographs of the experimental device where thermite melts through the steel target plate.

Fig. 2(a) shows a photograph of the overall experimental device set-up based on the schematic diagram in Fig. 1. The thickness of the shell is 2 mm. Fig. 2(b) shows that the distance between the charge port and steel target plate is about 3 cm.

Three sets of experiments were conducted for each material. The 6 cans of thermite were numbered 1–6. Of these, the odd numbers represent the stainless-steel material shell casing and the even numbers are the PVC material shell casing.

The group details are shown in Table 1.

**Table 1:** Thermite experiment numbers

No.	Shell material	Dose	Thermite	Shell height	Shell thickness
1	Stainless steel	200 g	1 μm Al	5 cm	2 mm
			40 μm MnO_2_		
2	PVC	200 g	1 μm Al	5 cm	2 mm
			40 μm MnO_2_		
3	Stainless steel	200 g	5 μm Al	5 cm	2 mm
			40 μm MnO_2_		
4	PVC	200 g	5μmAl	5 cm	2 mm
			40 μm MnO_2_		
5	Stainless steel	200 g	10 μm Al	5 cm	2 mm
			40 μm MnO_2_		
6	PVC	200 g	10 μm Al	5 cm	2 mm
			40 μm MnO_2_		

The 6 cans of thermite with different formulas in the experiments all had a charge weight of 200 g, and the other parameters, such as the height, diameter, and thickness of the shell, were the same.

To record the whole combustion process, a camera was placed about 5 m away from the experimental device, and this was used to photograph the burning remotely when the experiment started.

## 3. Theory and simulations

To explore the influence of the tubular shell material on the combustion properties of the thermite, a two-dimensional burning model was established. This was based on experiments where thermite melted through a steel target plate. During the process of building the model, the following assumptions were made:

(1) The combustion process involving flame-type thermite is equal-area combustion,^21^ which is complicated. However, the flame temperature and speed are the points of concern during the process of further exploration. Therefore, the combustion area is simulated simply as a time-varying heat source flowing with air. This zone is a dense airflow with a certain speed and density, similar to a flame.

(2) The heat transfer process includes heat conduction, convective heat transfer and heat radiation. Relevant respective models are established to analyze the heat transfer process.

(3) The process of convective heat transfer follows Newton's law of cooling.^22^

(4) The influence of the high-temperature melting endotherm of the PVC material is considered.

(5) Gravity is considered.

### 3.1 The fluid–solid heat transfer model

According to the laws of conservation of mass and energy, and considering heat-transfer dynamics and computational fluid dynamics,^23^ the following equations can be obtained:1
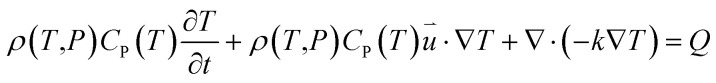
2
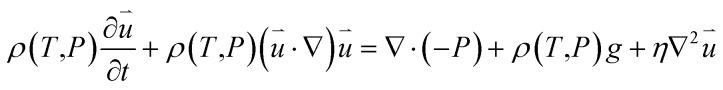
3
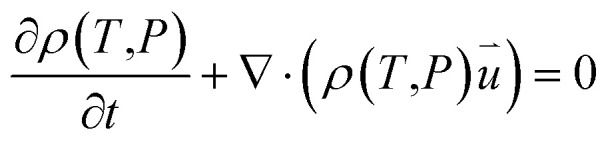
where *ρ*(*T*,*P*) is the material density (kg m^−3^) (since air density is greatly affected by temperature and pressure, the air–fluid density is a function of temperature and pressure), *C*_P_(*T*) is the constant pressure heat capacity (J (kg K)^−1^) (a function of temperature), *T* is temperature (K), 
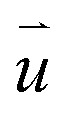
 is the particle velocity (m s^−1^), *P* is pressure (Pa), *Q* is the heat source (W), *k* is the heat transfer coefficient (W (m K)^−1^), *g* is acceleration due to gravity (m s^−2^), and *η* is the dynamic viscosity (Pa s).

At the initial moment (*t* = 0), the initial values are set as follows:
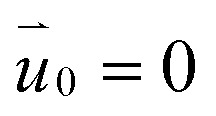
*P*_0_ = 1 atm*T*_0_ = 293.15 K

Open boundary conditions are adopted for the borders everywhere.4
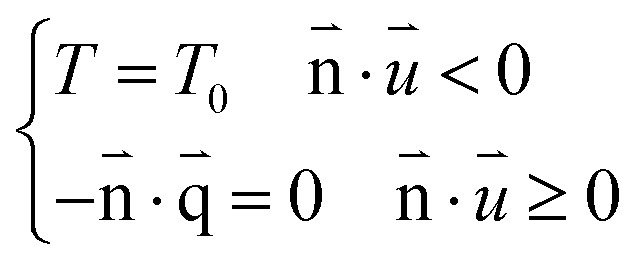
where 
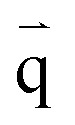
 is the heat flux (W m^−2^).

Open boundary conditions mean that the parameters at the boundary are not disturbed by the external environment and free exchange occurs with the environment.

The heat source term *Q* is defined as a dense airflow similar to a flame with a certain speed and density, in which the airflow temperature can reach up to 3000 °C.

### 3.2 The heat transfer process model

Studying the factors that affect the heat transfer efficiency during the entire heat transfer process is a main aspect of interest. In the overall heat transfer model (eqn (1)), the amount of heat transferred is calculated according to the following formula:^23^

5 *ϕ*_sum_ = −*kA*∇*T*

where *ϕ*_sum_ is the total heat transferred *via* heat transfer (W), *k* is the heat transfer coefficient (W (m K)^−1^), *A* is the area of the heat transfer interface during the experiment (m^2^), and ∇*T* is the spatial temperature difference (K).

The formula shows that the total amount (*ϕ*_sum_) of heat transfer affecting the entire process mainly depends on the heat transfer coefficient (*k*), which is related to the thermal conductivity of a material (*λ*) during heat conduction and the convective heat transfer coefficient (*h*) during convective heat transfer in this model.

### 3.3 Heat conduction

During the heat conduction process,^24^ the heat conduction can be modelled as shown in eqn (6):

6 *ϕ*_con_ = −*λA*_sol_∇*T*

where *ϕ*_con_ is total heat transferred during heat conduction (W), *λ* is the thermal conductivity (W (m K)^−1^), and *A*_sol_ is the contact area of the surface of the heat-conducting solid during the experiment (m^2^).

Eqn (6) shows that the main factor that affects the heat transfer efficiency of heat conduction is the thermal conductivity of the material, which affects the steel-target melting results directly. The thermal conductivity varies greatly between different materials, leading to great differences in the concentration and retention time of high temperatures during the burning and melting of thermite through steel targets.

### 3.4 Convective heat transfer

The convective heat transfer process follows Newton's cooling formula:^22^

7 *ϕ*_tro_ = *hA*_liq_(*t*_ext_ − *T*)

where *ϕ*_tro_ is the total heat transferred during convective heat transfer (W), *h* is the convective heat transfer coefficient (W (m^2^ K)^−1^), *A*_liq_ is the contact area of the fluid heat transfer surface during the experiment (m^2^), and *t*_ext_ is the fluid ambient temperature (K).

It can be seen from eqn (7) that the convective heat transfer coefficient directly affects the heat transfer efficiency during the process of convective heat transfer. However, the influencing factors are more complicated, and it is a variable that is closely related to the process.

To obtain the convective heat transfer coefficient, a layer close to the wall is used as a control surface, and the law of conservation of energy is applied to make the convective heat transfer equal the amount of heat close to the wall, as shown in eqn (8):8
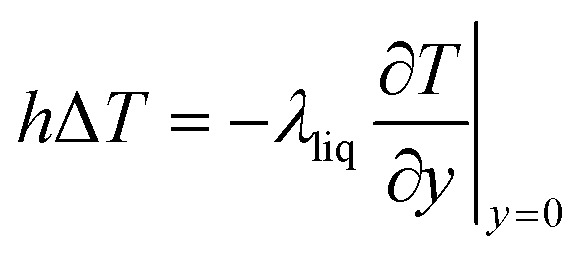
which takes the direction perpendicular to the solid–liquid interface as the *y*-direction, and the location infinitely close to the solid–liquid separation surface is set as *y* = 0. *λ*_liq_ is the thermal conductivity of the liquid (W (m K)^−1^).

Eqn (8) shows that although the process of solving for the convective heat transfer coefficient is more complicated, its relationship with the shell material is not obvious. Therefore, a difference in material does not affect the convective heat transfer coefficient.

### 3.5 Thermal radiation heat transfer

Due to the extremely high flame temperature, heat radiation may dominate the heat transfer process. The heat radiation heat transfer model^25^ is as shown in eqn (9):9
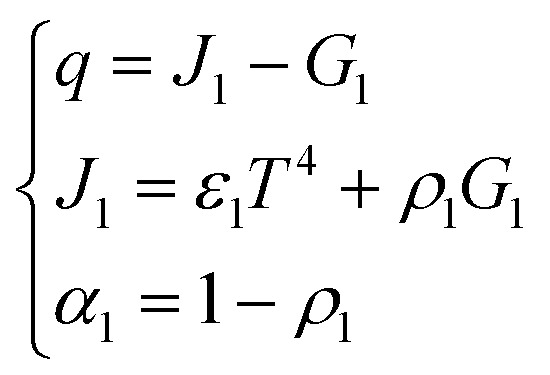
where *q* is the radiated heat per unit area (W m^−2^), *J*_1_ is the radiation energy leaving a unit of surface area per unit time (W m^−2^), *G*_1_ is the radiated energy reinvested per unit area of surface per unit time (W m^−2^), *ε*_1_ is the thermal emissivity, *T* is the object temperature, and *α*_1_ and *ρ*_1_ are the absorption ratio and reflection ratio, respectively.

**Fig. 3:**
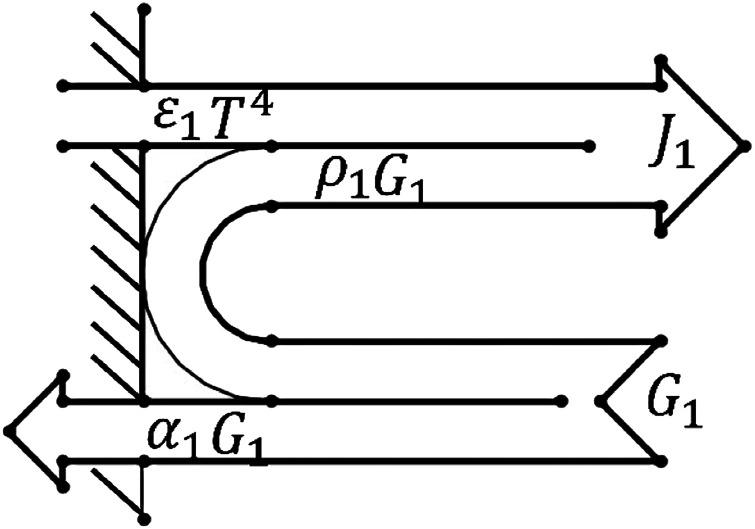
A schematic diagram of radiative heat transfer.

A schematic diagram of radiative heat transfer is shown in Fig. 3.

As shown in Fig. 3, the heat released through thermal radiation is related to *J*_1_ and *G*_1_, which are closely related to the absorption ratio *α*_1_ and reflection ratio *ρ*_1_ of the material.

Since the same heat source is always used, no matter what material the tubular housing is made from, it can be considered that the heat radiated by the heat source is the same. However, different shell materials have different absorption ratios and reflection ratios, which can cause different heat distributions.

Through theoretical analysis, it can be seen that differences in the thermal conductivities, thermal radiation absorption ratios, and reflection ratios of materials have a great influence on the heat transfer process. In experiments, these factors are significantly different between PVC and stainless steel. This series of factors will affect heat transfer during the heat transfer process, so that the flame distribution and duration of high temperature are greatly different and, eventually, the effects on the melting of the steel target plate will also be significantly different.

At the same time, the melting of PVC at high temperatures should also be considered, as this will absorb some of the heat. This will have a negative impact on the penetration of the steel plate, so the specific actual effect will depend on the results of experiments and simulations.

### 3.6 Numerical simulations

**Fig. 4:**
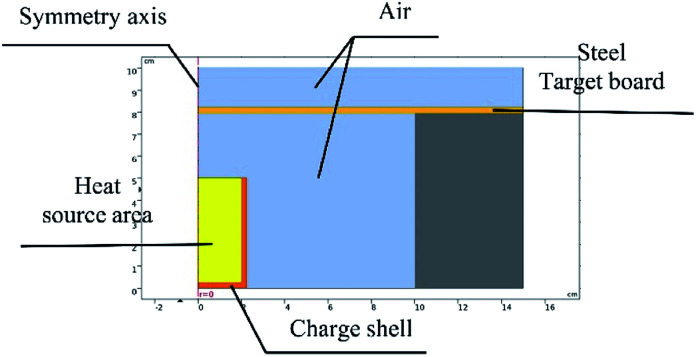
The structure used for simulation experiments.

Based on the experiments involving thermite melting through steel target plates, the model shown in Fig. 4 was established.

To reduce the number of simulation calculations and the calculation time while still ensuring accuracy, a half structure could be established for simulation experiments due to the symmetry of the experimental device. The parameter ratios were based on those used for practical experiments.

The model was simulated using the flow solid heat transfer module and laminar flow module in the software coupled with the non-isothermal flow module.

Table 2 lists the parameters of each material.^26^

**Table 2:** The material parameters used in the model^a^

Material	Thermal conductivity, W (m K)^−1^	Density, kg m^−3^	Constant pressure heat capacity, J (kg K)^−1^	Specific heat rate	Dynamic viscosity, Pa s	Reflectance	Melting temperature, °C	Melting heat, J kg^−1^
Air	*k*(*T*)	*ρ*(*p*_A_,*T*)	*C*_P_(*T*)	1.4	*η*(*T*)	—	—	—
A3 steel	44.5	7850	475	—	—	—	—	—
Stainless steel	76.2	7870	440	—	—	0.35	—	—
PVC	0.1	1760	1050	—	—	0.94	185–205	106 J kg^−1^

a *ρ*(*p*_A_,*T*) is the density of air, a function of temperature *T* and pressure *P*. *C*_P_(*T*) is the heat capacity of air at constant pressure, a function of temperature. *k*(*T*)is the thermal conductivity of air, a function of temperature.

The tubular shell materials are stainless steel and PVC, respectively. Although the parameters of the materials, such as density and constant pressure heat capacity, are different, according to theoretical analysis, only differences in the thermal conductivities and the heat radiation correlation coefficients play a key role in this model and the experiments.

## 4. Results and discussion

### 4.1 Analysis of the experimental results

The experimental process was recorded using a camera. In comparison experiments involving the three groups, the experimental phenomena were similar, so one group of experimental results was selected for further analysis.

**Fig. 5:**
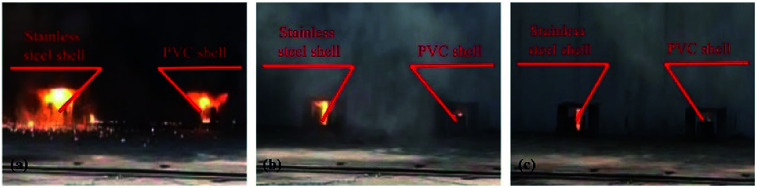
Comparative experimental images of shells at different moments: (a) about 0.5 s, (b) about 1.5 s, and (c) about 2.5 s after ignition.

Images are shown in Fig. 5.

Fig. 5 compares the combustion processes of thermite in different tubular shell materials through the steel target plates from the same group. The left side of each image shows thermite constrained by the stainless-steel shell material and the right side shows the PVC shell material.

As shown in Fig. 5(a), after burning for about 0.5 s, it is observed that the thermite burning processes are both extremely violent, producing bright flames. However, the flame from thermite constrained by the stainless-steel material is brighter and more dispersed in space, almost filling the gap between the thermite and the steel target plate. To the contrary, the brightness of the thermite with the PVC material shell is slightly weaker, but the flame is more concentrated. After burning for about 1.5 s, as shown in Fig. 5(b), both fires are almost completely extinguished, however, the stainless-steel tube body is red and shiny. After about 2.5 s, as shown in Fig. 5(c), both reactions are complete. The red metal of the stainless-steel pipe body can still be seen, however, the thermite with PVC material shows almost no remaining effects.

The reason for this is that the thermal conductivity of the stainless-steel material is much higher than that of the PVC material, and the heat radiation absorption ratio is high and the reflection ratio is low; therefore, under high-temperature conditions, heat and temperature are quickly transferred to the entire pipe body, rather than being easily lost, resulting in a bright red phenomenon being seen.

**Fig. 6:**
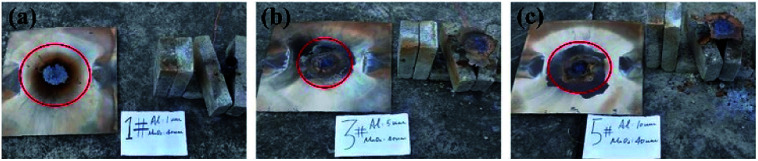
The effects of thermite acting on the steel target plates under the restraint of stainless-steel tubes.

**Fig. 7:**
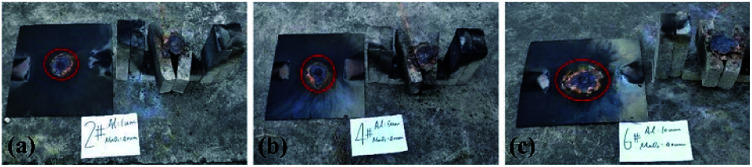
The effects of thermite acting on the steel target plates under the restraint of PVC.

It can be seen from Fig. 5 that under the constraints of different materials, the thermite burning phenomena are different. After the reaction area finished cooling, the steel target plates were turned over and observed. The combustion effects on the sides facing the flame are shown in Fig. 6 and 7.

Fig. 6 shows the effects of thermite acting on steel target plates under the stainless-steel tube restraint, and Fig. 7 presents the PVC material effects. In each picture, the part surrounded by the red circle is clearly different from the other areas of the steel target plate. The reason for this phenomenon is that after this area is burned by the thermite, the corresponding steel target plate does not melt all the way through at this temperature; it then returns to a normal temperature and cools down, and this area is formed. We call this area the molten region.

The steel target plates related to thermite restrained by stainless-steel tubes shown in Fig. 6 have much larger melting areas than the steel target plate related to thermite restrained by PVC tubes shown in Fig. 7. During the experiments, it is found that combustion is extremely rapid, and the whole process only lasts for 1–2 s. Therefore, the reason for the above phenomenon is probably related to the degree of flame spreading.

**Fig. 8:**
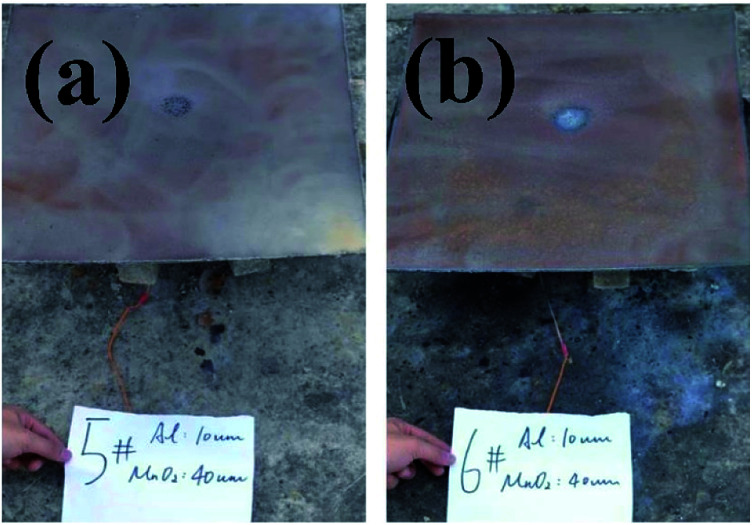
Rear views of steel target plates under the effects of thermite constrained by different tube bodies: (a) stainless steel; (b) PVC.

The backs of the steel target plates no. 5 and no. 6 were then used for comparison, as shown in Fig. 8. Fig. 8(a) is target plate no. 5, and Fig. 8(b) is target plate no. 6.

The side of steel target plate no. 5 not facing the flame just reveals a molten area, showing that the melting effect was not effective. However, the melting effect on steel target plate no. 6 is greatly prominent, almost melting through the steel target plate. This result shows that the use of thermite under the PVC pipe restraint is better than under the stainless-steel pipe.

### 4.2 Analysis of the simulation results

**Fig. 9:**
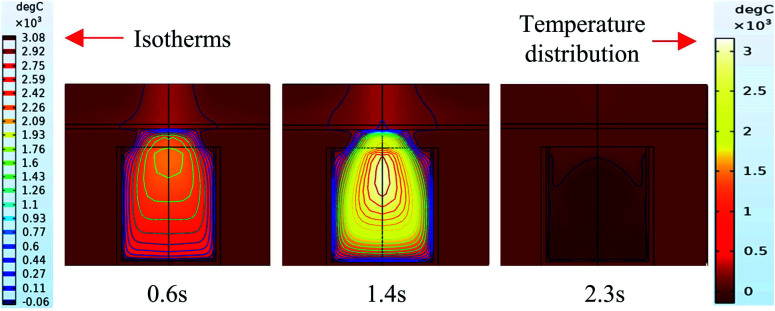
Simulation of the effects of thermite acting on the steel target under the constraint of stainless steel.

**Fig. 10:**
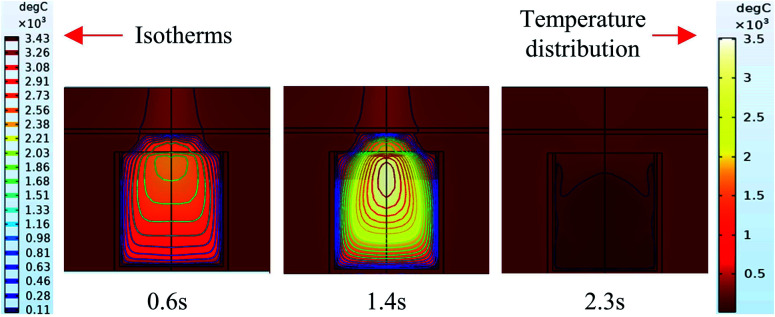
Simulation of the effects of thermite acting on the steel target under the constraint of PVC.

It can be seen from the experiments that the entire combustion process is extremely rapid, and the reaction was completed in about 2.5 s. Fig. 9 and 10 show state diagrams of thermite under the constraints of stainless-steel and PVC pipe bodies, respectively, 0.6 s, 1.4 s, and 2.3 s after ignition.

During the simulations, the same heat source is used. Fig. 9 and 10 show the isotherms and temperature distributions 0.6 s, 1.4 s, and 2.3 s after the combustion of thermite. The isotherms and different colors in different areas represent different temperatures. The isotherm and temperature distribution legends detailing the corresponding colors are given on the left and right sides of the images, respectively.

From the temperature distributions in different areas, it can be observed that the highest temperature that can be reached by the thermite constrained by the PVC pipe body is nearly 500 °C higher than that constrained by the stainless-steel pipe body, and the temperature under the constraint of the PVC pipe body material is higher than the temperature in the same space of the stainless-steel-constrained thermite. From the distribution of the isotherms, it can be found that the isotherm distribution of the thermite constrained by the stainless-steel tube has a wide and sparse diffusion range, especially 1.4 s after combustion. This shows that during the burning of thermite, the temperature of the stainless-steel shell material diffuses faster, along with a wider distribution of the flame in space, which corresponds with the phenomenon shown in Fig. 5(a), where the fire almost fills the gap between the charge and the steel target plate.

**Fig. 11:**
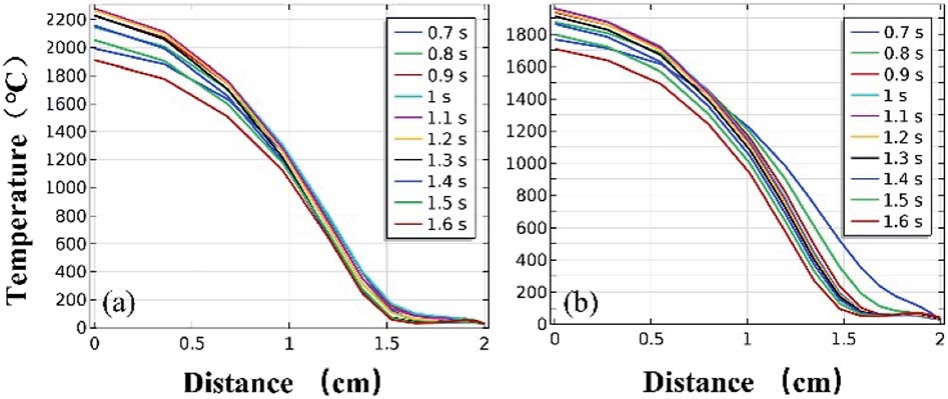
Simulated temperature distributions at the charge ports of the different material shells: (a) PVC; (b) stainless steel.

**Fig. 12:**
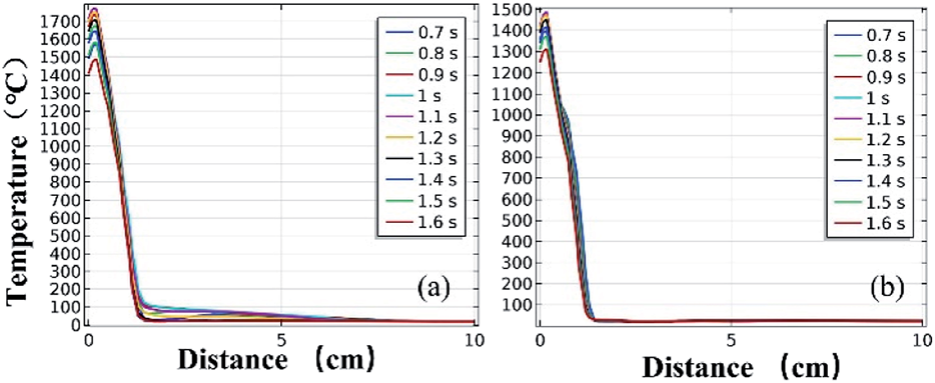
Simulated temperature distributions of the steel plates above the different material shells: (a) PVC; (b) stainless steel.

Fig. 11 and 12 further show the temperature distributions at the charge ports and surfaces of the steel target plates. Fig. 11 shows a simulation of the temperature distributions at the charge ports for the two different materials, and Fig. 12 shows the temperature distributions at the steel target plates for the two different materials. In Fig. 11 and 12, (a) corresponds to the PVC material shell, and (b) corresponds to the stainless-steel material. The curves of different colors represent the temperature distributions at different moments, and only the period from 0.7–1.6 s is sharply reactive during the selected processes.

It can be clearly seen from Fig. 11 and 12 that the temperature of the PVC pipe material is higher than that of stainless steel, regardless of whether the temperature distribution at the charging port or the temperature distribution on the surface of the steel target plate is considered; the maximum temperature of PVC can reach 2200 °C at the charging port and 1700 °C at the steel plate, while those of the stainless-steel shell material are 1800 °C and 1500 °C.

The thermite in the PVC tubular shell remains at a temperature above 1500 °C, which is the melting point of the steel target plate, for a longer period of 0.9 s. Therefore, it appears that the steel target plate will almost melt, but only a melting zone is produced, as the reaction time is too short; this is consistent with the experimental results.

According to the above theoretical analysis, on one hand, the thermal conductivity of the PVC material is much lower than that of stainless steel. The lower thermal conductivity and heat loss make it easier to maintain a high temperature at the loading port, so the heated area of the steel target plate is more concentrated, thus melting the steel target plate more.

On the other hand, from the perspective of radiative heat transfer, it goes without saying that the heat radiation is alike because the same heat source is used. The heat radiation reflectance coefficient of the PVC pipe material is a little higher than that of stainless steel. When heat from the heat source is radiated onto the PVC pipe body material, the material can reflect more heat, which causes more heat to be collected in the pipe body. This further proves that when PVC is used as the pipe body material, more heat can be concentrated, and the temperature distribution is denser.

It is believed that PVC materials will melt and absorb heat at high temperatures. Compared to stainless steel, PVC will melt and absorb a lot of heat, but the heat lost from conduction and heat radiation is much less than in the case of stainless steel. Therefore, in summary, a PVC tubular shell is more conducive to temperature concentration than stainless steel, and is more conducive to melting through steel plates, with similar experimental and simulation results.

## 5. Conclusions

This paper has explored the impact of tubular shell materials on thermite combustion effects from experimental design and theoretical analysis aspects, and software has been used for simulation calculations.

The experimental results showed that the effect of the PVC material on the melting through of the steel target plate was obvious, while thermite constrained by the stainless-steel tube only produced a melting zone. The results demonstrated that thermite with a PVC pipe body can penetrate the steel target plate better.

Based on the experimental design, a fluid–solid heat transfer model was established and analyzed. Through analysis, we could see that the thermal conductivity and coefficient of thermal radiation of the material played crucial roles in determining the steel plate melting behavior. The smaller the thermal conductivity of the material and the greater the thermal radiation reflectance coefficient, the more obvious the effects on the steel target plate.

Software was used to simulate the model. From the simulation results, thermite with PVC material as the shell could generate a higher temperature that lasted for longer. The temperature at the charging port could reach a maximum of 2200 °C with a maximum of 1700 °C at the steel target plate, which was held for about 0.9 s. The melting point of the steel target plate was about 1500 °C, so it was almost melted through. On the contrary, the maximum temperatures of the stainless-steel shell material were 1800 °C and 1500 °C, respectively, which could not produce a melt-through effect.

The simulation results are consistent with the experimental results, and the theoretical model has certain guiding significance for experimental explorations. Although PVC melting and heat absorption will have negative effects on the penetration of the steel plate, its thermal conductivity and thermal radiation coefficient play more critical roles here, and the positive effects exceed the melting heat absorption. These results are of guiding significance for the future design, performance improvement, and theoretical study of thermite-shell combustion processes.

## Conflicts of interest

The authors declare that there are no conflicts of interest regarding the publication of this paper.

## Supplementary Material
